# Anti-tumor effect of β-glucan from *Lentinus edodes* and the underlying mechanism

**DOI:** 10.1038/srep28802

**Published:** 2016-06-29

**Authors:** Hui Xu, Siwei Zou, Xiaojuan Xu, Lina Zhang

**Affiliations:** 1 College of Chemistry and Molecular Sciences, Wuhan University, Wuhan 430072, China

## Abstract

β-Glucans are well known for its various bioactivities, but the underlying mechanism has not been fully understood. This study focuses on the anti-tumor effect and the potential mechanism of a branched β-(1, 3)-glucan (LNT) extracted from *Lentinus edodes*. The *in vivo* data indicated that LNT showed a profound inhibition ratio of ~75% against S-180 tumor growth, even significantly higher than the positive control of Cytoxan (~54%). Interestingly, LNT sharply promoted immune cells accumulation into tumors accompanied by cell apoptosis and inhibition of cell proliferation during tumor development. Furthermore, LNT not only up-regulated expressions of the tumor suppressor p53, cell cycle arrestin p21 and pro-apoptotic proteins of Bax and caspase 3/9, but also down-regulated PARP1 and anti-apoptotic protein Bcl-2 expressions in tumor tissues. It was first found that LNT initiated p53-dependent signaling pathway to suppress cell proliferation *in vitro*, and the caspase-dependent pathway to induce cell apoptosis *in vivo*. The underlying anti-tumor mechanism was proposed that LNT activated immune responses to induce cell apoptosis through caspase 3-dependent signaling pathway and to inhibit cell proliferation possibly via p53-dependent signaling pathway *in vivo*. Besides, LNT inhibited angiogenesis by suppressing VEGF expression, leading to slow progression of tumors.

Cancer is a universal health problem with high morbidity and mortality, constituting an enormous burden on society in more and less economically developed countries alike. Based on GLOBOCAN estimates, ~14.1 million new cancer cases and 8.2 million deaths occurred in 2012 worldwide^1^. Cancer is also currently the second leading cause of death in the United States, and is expected to surpass heart diseases as the leading cause of death in the next few years^2^. Conventional cancer therapies, such as surgery, chemotherapy and radiotherapy, are showing various limitations because of poor prognosis and serious side effects^3^. Therefore, it is urgent to search for effective anti-cancer novel drugs with high safety to prevent or treat the increasing cancer cases throughout the world every year.

In past decades, polysaccharides isolated from natural sources, such as fungi, plants, algae and animals, have been widely studied and indicated to show anti-tumor activity^4^. Unlike existing small molecular anti-cancer chemicals, polysaccharides are well known to have no toxic side effects. Several polysaccharides such as PSK and schizophyllan are currently used clinically as anti-tumor agents^5^,^6^ mainly in Japan, but have not been used worldwide. When used as cancer therapeutics, these polysaccharides are able to significantly prolong the life span of cancer patients and really improve their quality of life^7^,^8^. Therefore, polysaccharides are a good candidate for cancer therapy. In particular, Lentinan (LNT), a β-1, 3-glucan with two β-1, 6-glucose branches every five glucose residues in the backbone from *Lentinus edodes*^9^, as the first medicinal macrofungus to enter the realm of modern biotechnology^10^, is especially striking for its anti-tumor activity possibly due to its unique triple helical conformation^11^. In our previous work, we have demonstrated that LNT can stimulate macrophage-like RAW264.7 cells through activation of mitogen-activated protein kinases (MAPKs) and nuclear transcription factor NF-κB p65^12^. Moreover, LNT shows strong inhibition against lipopolysaccharide-induced inflammation through suppressing phosphorylation of MAPKs^13^. As is known, the molecular mechanism of anti-tumor activity of drugs is critical for clinical trials. Therefore, there are many researches on antitumor activity and mechanism of action for polysaccharides including Lentinan^14^,^15^,^16^,^17^,^18^,^19^,^20^,^21^,^22^. For example, Chihara *et al*. reported that Lentinan caused complete regression of Sarcoma 180 (S-180) cells transplanted into mice at a dose of 1 mg/kg for 10 days, while a large dose of 80 mg/kg for 5 days showed no antitumor activity in comparison with the untreated control mice^14^,^15^. The anti-tumor mechanism was summarized to improve immune function^16^,^17^,^18^,^19^, induce apoptosis of tumor cells^20^,^21^, increase NK and T helper cells and stimulate the synthesis of interleukins^22^. However, to our best knowledge, the mechanism of anti-cancer/tumor for Lentinan is still preliminary, and the details on signaling pathways have not been completely revealed, which may become one of the major problems limiting its application worldwide.

Based on the above, in this study, we performed *in vivo* (mice sarcoma S-180 tumor model) and *in vitro* (S-180 and human cervical carcinoma Hela cells) experiments to explore the potential mechanism of anti-tumor by using confocal microscopy, western blot, histology and immunohistochemical staining, and immunofluorescence staining etc. For the first time, we found that LNT could directly interact with tumor cells for initiating p53-dependent pathway to suppress tumor cell proliferation but showed no cytotoxicity against normal cells *in vitro*. The *in vivo* data demonstrated that LNT showed remarkable anti-tumor effect through activating immune cells to promote tumor cell apoptosis via caspase-dependent signaling pathway, and to inhibit tumor cell proliferation possibly via p53-dependent pathway. Our results will provide a better understanding of anti-tumor action for β-glucans.

## Results

### LNT shows significant inhibition against S-180 tumor growth in mice

**Figure 1:**
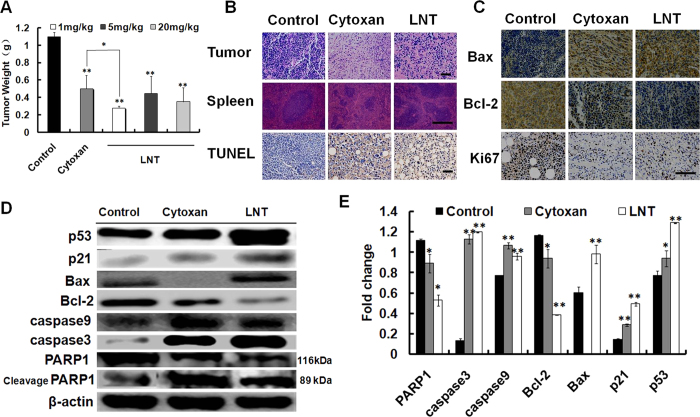
Effects of LNT on S-180 tumor cells apoptosis and proliferation *in vivo*. (**A**) Effect of LNT on S-180 tumor weight in mice, **p < 0.001 versus the control, *p < 0.05 versus cytoxan. All data *in vivo* are expressed as means ± SD of three independent experiments, and each performed in duplicate. The data *in vivo* are expressed as means ± SD of 10 mice in each group. (**B**) Histological analysis by hematoxylin and eosin staining (H&E) of sections of tumors and spleens from mice, and TUNEL staining of tumors. Scale bars, 25 μm. (**C**) Immunohistochemical staining for apoptotic cells (Bax and Bcl-2 staining) and proliferating cells (Ki67 staining) in tumor tissue with positive staining for the biomarker shown. Scale bars, 50 μm. (**D**,**E**) S-180 tumor tissue proteins were extracted and p53, p21, Bax, Bcl-2, caspase 3, caspase 9 and PARP1 were detected by western blot analysis using their specific antibodies with β-actin as the loading control. The blots are run under the same experimental conditions. Experiments were performed at least twice, and protein expression was determined by quantitative densitometry. The results shown are representative of three independent experiments. *p < 0.05 and **p < 0.001 versus the control.

Although sarcomas are relatively rare malignant tumors comprising less than 10% of all cancers^23^, they affect ~11,000 individuals in the United States and ~200,000 individuals worldwide each year^24^. Therefore, S-180 tumor cells were chosen to investigate the effect of LNT on tumor growth in mice with cyclophosphamide (Cytoxan, 20 mg/kg per day) as the positive control. As a result, LNT at different dosages of 1 mg/kg, 5 mg/kg and 20 mg/kg markedly protected mice against tumor development in contrast to the negative control as shown in Fig. 1A. In particular, LNT at the dosage of 1 mg/kg showed higher inhibition against tumor than the positive control of Cytoxan with statistically significant difference, suggesting the striking anti-tumor activity of LNT. Table 1 summarized all the data including inhibition ratios, enhancement ratios of body weights, spleen and thymus indexes. Clearly, no significant changes in spleen and thymus indexes were observed in LNT-treated mice compared with the negative control, showing the good safety of LNT, which was further confirmed by H&E staining of spleen sections with the similar lymph nodes density in the control and LNT-treated mice (Fig. 1B, spleen panel). However, the two indexes significantly decreased in Cytoxan-treated group, indicative of the strong cytotoxicity of Cytoxan. Histological evaluation of H&E staining of tumor sections showed that the nuclear pycnosis and rupture occurred in LNT-treated and Cytoxan-treated mice but not in the control (Fig. 1B, tumor panel). It is thus conclude that LNT is a good drug candidate to treat solid tumors with low toxic side effect. As shown in Fig. 1A and Table 1, the anti-tumor effect of LNT at the three dosages showed no significant difference, and the following experiments on anti-tumor mechanism were thus performed only for the relatively high inhibition ratio at the dosage of 1 mg/kg.

### LNT promotes apoptosis and inhibits the proliferation of S-180 tumor cells in mice

The immunohistochemistry results indicated that LNT induced tumor cell apoptosis indicated by an increase of the TUNEL-positive cell numbers (Fig. 1B, TUNEL panel) and Bax expression (Fig. 1C, Bax panel), and a decrease of Bcl-2 expression (Fig. 1C, Bcl-2 panel). Additionally, LNT induced a significant reduction in Ki67 staining (Fig. 1C, Ki67 panel), indicating that LNT significantly reduced tumor cell proliferation^25^. Therefore, it can be concluded that LNT inhibits tumor growth through inducing cell apoptosis and suppressing cell proliferation.

It has been reported that tumor suppressor genes are needed to regulate the cycle of cell growth, DNA replication and division into two new cells for keeping cells under control^26^, which are strongly associated with the cell proliferation. Thereinto, p53 is an important tumor suppressor that either counters cell proliferation or more dramatically induces various cell cycle checkpoints, apoptosis or cellular senescence^26^. To see whether LNT affected the expression of p53 or not, proteins in tumor tissues were extracted and analyzed by western blot. As shown in Fig. 1D,E, a sharp up-regulation of p53 in LNT-treated mice was observed. Interestingly, expressions of p21 and Bax, both of which are directly regulated by p53^26^, were also enhanced, indicating that LNT inhibited S-180 tumor growth possibly through blocking cell proliferation and inducing cell apoptosis or cellular senescence via targeting p53. A remarkable down-regulation of the anti-apoptotic Bcl-2 level was also seen in Fig. 1D,E. Besides, LNT induced up-regulation of caspase 9 and 3 expressions, implying that LNT possibly induced apoptosis via the traditional nuclear DNA damage pathways^27^. As we know, poly [ADP (ribose)] polymerase (PARP1) helps cells to maintain their viability, cleavage of which facilitates cellular disassembly and serves as a marker of cells undergoing apoptosis^28^,^29^. Therefore, the increased cleavage of PARP1 by caspase 3 *in vivo*^30^ (Fig. 1D,E) confirmed the occurrence of an earlier mitochondria-related apoptosis through the caspase-dependent signaling pathway triggered by LNT.

Taken together, LNT inhibited S-180 tumor growth by promoting cell apoptosis and suppressing cell proliferation through caspase- and p53-dependent signaling pathways.

### LNT induces infiltration of immune cells and cytokines/chemokines production in S-180 tumor tissues

**Figure 2:**
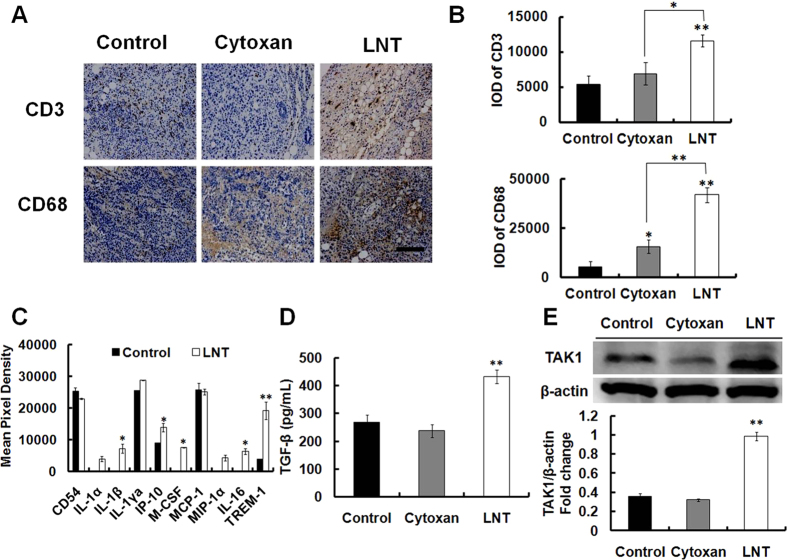
LNT induces infiltration of immune cells and cytokines/chemokines production in S-180 tumor tissues. (**A**) Immunohistochemistry of tumor tissue, stained with antibody against CD3 and CD68 (brown). Scale bars, 25 μm. (**B**) Quantification (IOD) of CD3 and CD68 per 100 mm^2^ of (**A**), *p < 0.05 and **p < 0.001 versus the control or cytoxan. (**C**) Cytokines in S-180 tumor tissue lysates by mouse cytokines array. *p < 0.05 and **p < 0.001 versus the control. (**D**) TGF-β in tumor lysates was assayed by ELISA, **p < 0.001 versus the control. (**E**) TAK1 expression in S-180 tumor tissues was detected by western blot using its specific antibody. The blots are run under the same experimental conditions. The results shown are representative of three independent experiments, **p < 0.001 versus the control.

By immunohistochemical staining analysis, it was observed that CD68 (a marker of macrophages) and CD3 (a marker of T cells) expressions in tumor tissues from LNT-treated mice were much higher than those in the control and Cytoxan groups (Fig. 2A,B), indicating that LNT triggered strong immune responses to induce infiltration of immune cell populations to the tumor microenvironment. It is possible that LNT repressed tumor growth through activating immune responses. Since there were immune cell populations infiltrated into the tumor tissues, cytokines and chemokines in the tumor tissues were performed using a Proteome profiler array. As a result, enhancements of IL-1β, IP-10, M-CSF and TREM-1 levels were observed in tumor tissues from LNT-treated mice (Fig. 2C), further confirming the infiltration of immune cells to the tumor microenvironment^31^,^32^.

Transforming growth factor beta (TGF-β) has been shown to promote apoptosis significantly contributing to the tumor suppression during carcinoma initiation and progression^33^. In present, many genetic and pharmacological approaches have been used to define clear roles for TGF-β in cancer, and finally to develop highly specific strategies for targeted therapeutic intervention. In this work, the tumor was at the stage of initiation and progression because the duration time was stopped on Day 10. The levels of TGF-β in tumor lysates from LNT-treated mice were thus determined and found to rise significantly (Fig. 2D), suggesting occurrence of cell apoptosis. Moreover, the expression of TGF-β- activated kinase 1 (TAK1) was also seen to be up-regulated by LNT (Fig. 2E), indicating immune responses induced by LNT^34^.

**Figure 3:**
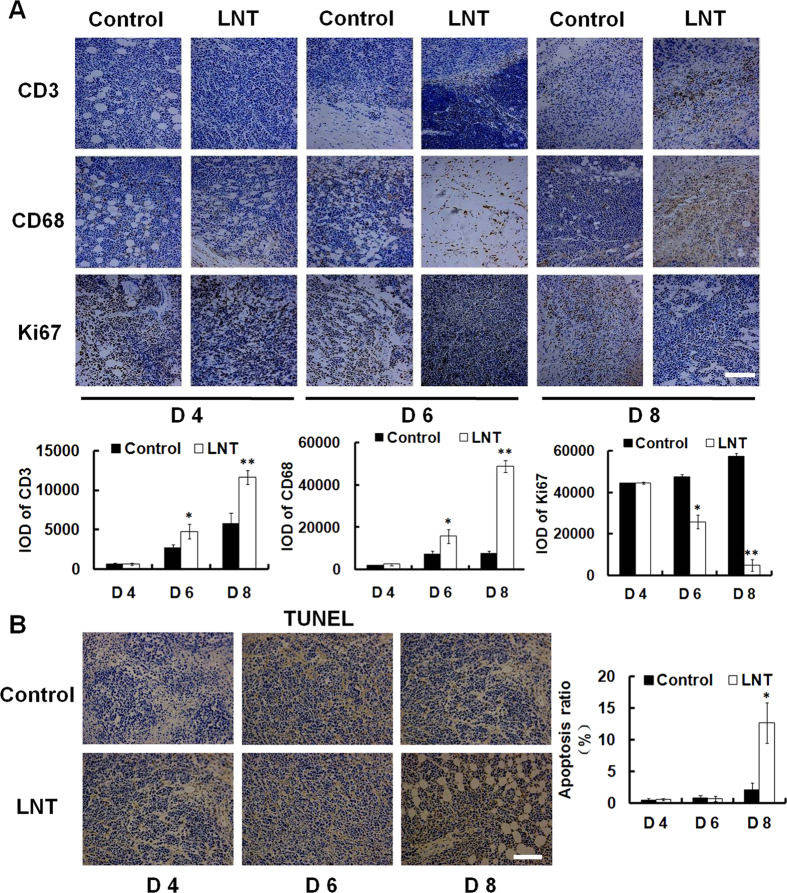
Effect of LNT on S-180 tumors at different time point. (**A**) Immunohistochemical analysis of CD3, CD68 and Ki67 in tumor tissues on day 4, day 6 and day 8, and the quantification of IOD per 100 mm^2^. (**B**) TUNEL assay of tumor tissues on day 4, day 6 and day 8. Brown is the positive staining, and apoptosis ratio is estimated as positive cells/total cells. Scale bars, 50 μm. *p < 0.05, **p < 0.001 versus the control.

Figure 3 shows the results of immunohistochemical staining analysis for tumor tissues from mice treated with LNT for different time within 10 days. Clearly, LNT significantly increased CD3 and CD68 expressions of tumor tissues on day 6 and day 8, and inhibited tumor cell proliferation from day 6 (Fig. 3A, Ki67 staining). The TUNEL staining results showed cell apoptosis occurred on day 8 but not on day 6 (Fig. 3B). Moreover, tumor growth was significantly reduced, and the inhibition ratio reached ~70% on day 6 (Fig. S1). All these results demonstrated that LNT activated immune responses to suppress tumor cell proliferation, and to promote cell apoptosis during tumor progression.

### LNT suppresses angiogenesis and Stat 3 activation in S-180 tumor tissues

**Figure 4:**
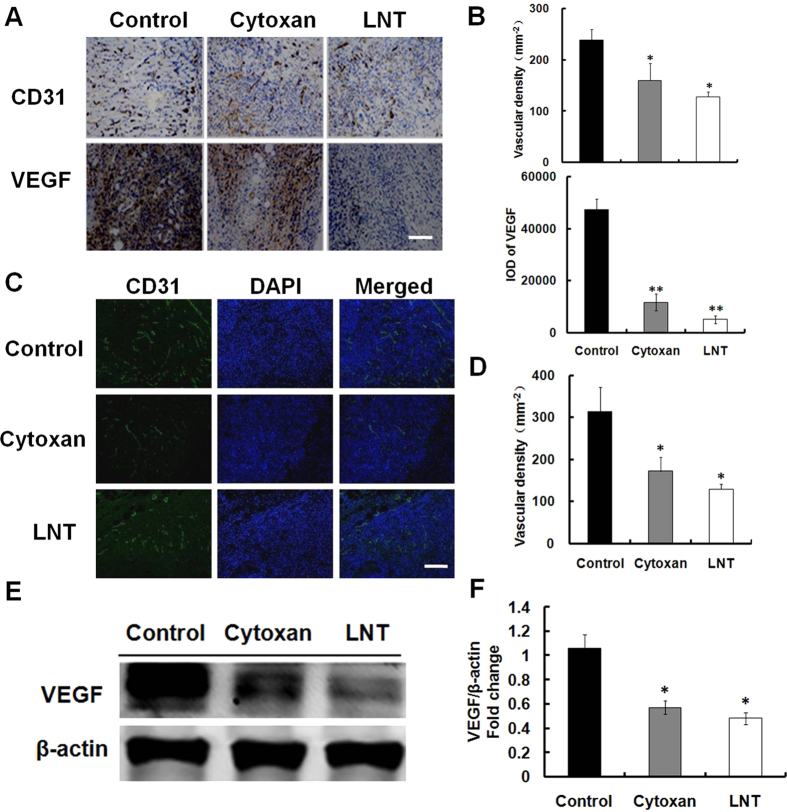
LNT inhibits angiogenesis in S-180 tumors. (**A**) Immunohistochemical analysis of CD31 and vascular endothelial growth factor (VEGF) in S-180 tumors. Scale bars, 25 μm. (**B**,**D**) Quantification of MVD as mean number of vessels per 100 mm^2^, *p < 0.05 and **p < 0.001 versus the control. (**C**) Immunofluorescence assay of CD31. (**E**) Western blot analysis of tumor protein extracts to detect VEGF using its specific antibody. The blots are run under the same experimental conditions. (**F**) The expression of VEGF was determined by quantitative densitometry. *p < 0.05 versus the control. The images and western blot results shown are representative of three independent experiments.

Anti–angiogenesis has been reported to be another mechanism of antitumor^35^. The microvasculature density (MVD) of the tumor was thus measured by immunohistochemical staining of CD31, a marker of endothelial cells (both established and nascent). Tumors from LNT-treated mice displayed reduced MVD compared with control tumors (Fig. 4A). Image analysis by counting CD31 positive vessels revealed significant differences in tumor microvessel density between the control and LNT-treated groups (Fig. 4B). The expression of CD31 was also determined by immunofluorescence, and the results were consistent with the immunohistochemistry analysis (Fig. 4C,D). The decreased expression of angiogenic protein VEGF in tumor lysates further confirmed anti–angiogenesis in LNT-treated mice (Fig. 4E), consistent with immunohistochemistry data (Fig. 4A). Overall, LNT treatment decreased the microvessel number and new microvessels formation.

**Figure 5:**
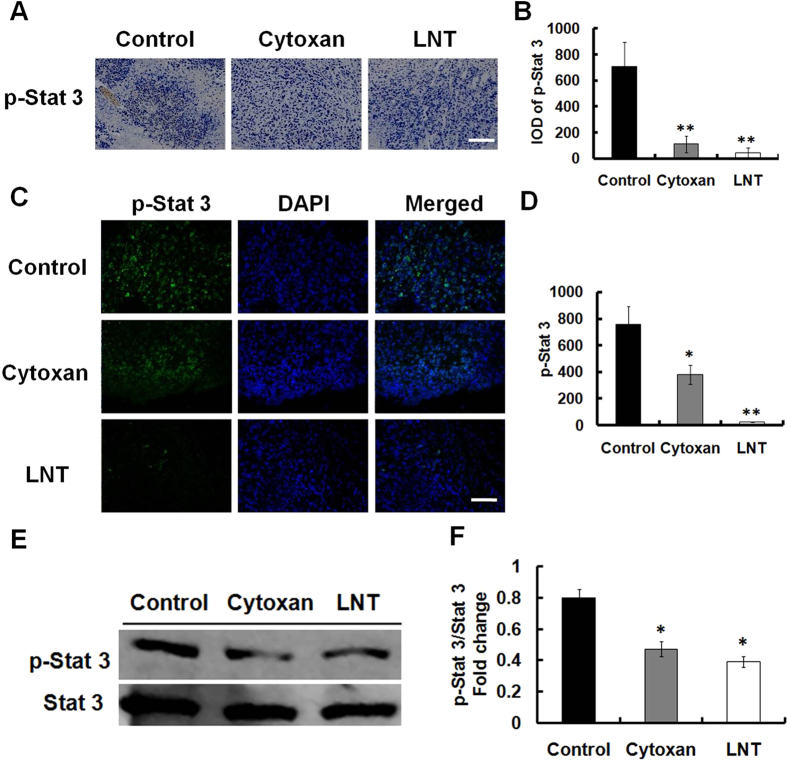
LNT suppresses Stat 3 activation in S-180 tumor tissues. Immunohistochemical analysis (**A**) and its quantification of IOD per 100 mm^2^ (**B**) of p-Stat 3 in tumors. Scale bars, 25 μm. *p < 0.05, **p < 0.001 versus the control. Immunofluorescence assay (**C**) and its quantification of IOD per 100 mm^2^ (**D**) of p-Stat 3. Scale bars, 25 μm. (**E**) Western blot analysis of phospho-Stat 3 and total Stat 3 in protein extracts prepared from tumors (representative of three experiments) using their specific antibodies. The blots are run under the same experimental conditions. (**F**) The digital results of Stat 3 and p-Stat 3 were determined by quantitative densitometry. *p < 0.05 versus the control. The image and western blot results shown are representative of three independent experiments.

Stat 3 phosphorylation plays a critical role in proliferation and survival of tumor cells, and angiogenesis in tumor tissues^36^. Accordingly, we evaluated the effect of LNT on constitutive p-Stat 3 levels in tumor tissues by immunohistochemical and immunofluorescent analysis. As a result, LNT substantially inhibited the constitutive Stat 3 activation in LNT-treated mice compared with the control (Fig. 5A–D). Western blotting analysis of phosphorylated Stat 3 (ser727) and total Stat 3 showed that tumor samples in the control contained more phosphorylated Stat 3 (ser727) than the LNT-treated group without change of total Stat 3 protein levels (Fig. 5E,F). These results implied that LNT-induced suppression of Stat 3 phosphorylation in tumors might contributed to inhibition of S-180 tumor cell proliferation and angiogenesis^36^.

### LNT inhibits cell proliferation/viability of S-180 and Hela cells *in vitro*

**Figure 6:**
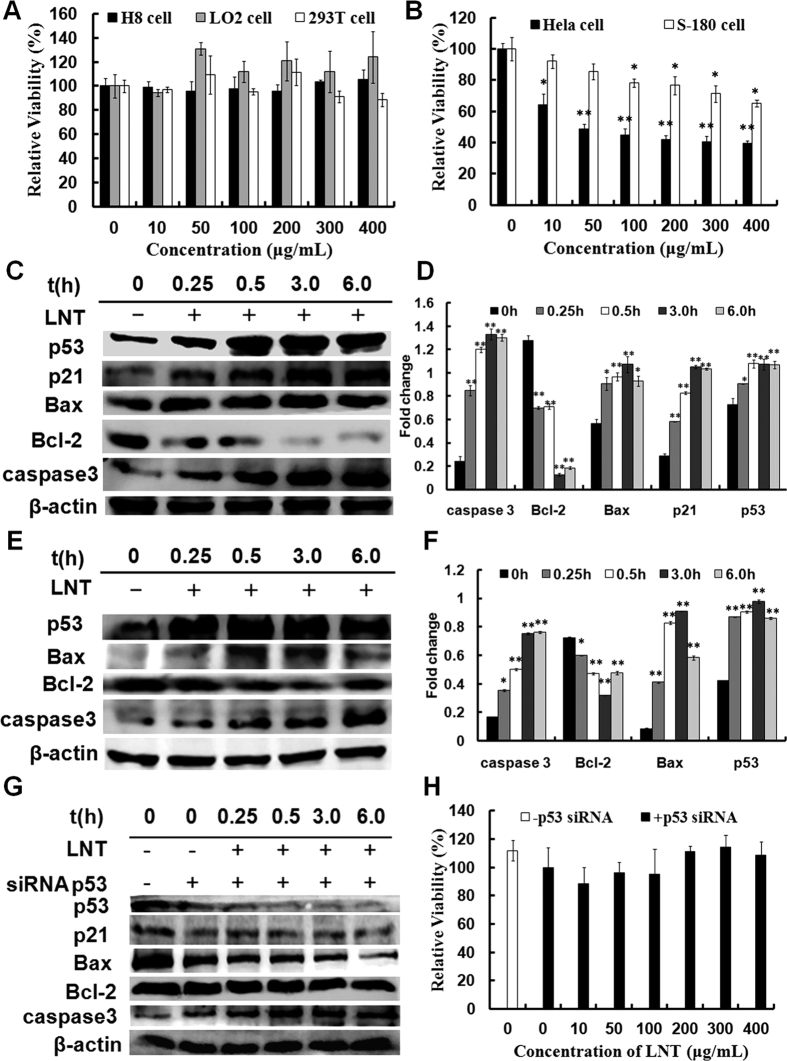
Effect of LNT on cell viability and apoptosis in Hela and S-180 tumor cells. **(A**) Cell viability of H8, LO2 and 293T cells after treatment with LNT for 48 h determined by MTT assay. (**B**) Cell viability of Hela and S-180 cells after treatment with LNT for 48 h determined by MTT assay. *p < 0.05 and **p < 0.001 versus the control (0 μg/mL). Hela (**C**) and S-180 (**E**) cells were respectively treated with LNT (200 μg/mL, 400 μg/mL) for indicated time intervals, and the whole cell extracts were prepared, 30 μg of which were resolved on 10% or 12% SDS-PAGE. p53, p21, Bax, Bcl-2 and caspase 3 were detected by western blot analysis using their specific antibodies with β-actin as the loading control. (**D**,**F**) The digital results were determined by quantitative densitometry. The results shown are representative of three independent experiments. *p < 0.05 and **p < 0.001 versus the control. (**G**) Hela cells were transfected with p53 siRNA, and then treated with LNT (200 μg/mL) for indicated time intervals, and the whole cell extracts were prepared, 30 μg of which were resolved on 10% or 12% SDS-PAGE. p53, p21, Bax, Bcl-2 and caspase 3 were detected by western blot analysis using their specific antibodies with β-actin as the loading control. The blots are run under the same experimental conditions. (**H**) Hela cells were transfected with p53 siRNA, and then treated with or without LNT at desired concentration for 48 h. Cell viability was determined by MTT assay.

As shown above, activation of many signal molecules was observed in S-180 tumor tissues, which was possibly associated with repression of tumor growth. To clearly clarify the anti-tumor mechanism, the *in vitro* test was performed. Methyl thiazolyl tetrazolium (MTT) assay is a classical method to assess the cell proliferation/viability *in vitro*. Therefore, the effects of LNT on cell proliferation and viability *in vitro* were first performed by MTT assay. As shown in Fig. 6A, LNT showed no visible effect on cell viability of the normal cells including H8, LO2 and 293T. However, the cell viabilities of S-180 and Hela tumor cells were repressed by LNT in a dose-dependent manner. In particular, LNT showed higher inhibition of Hela cell viability, which decreased to lower than 50% at the dosages of 50 μg/mL (Fig. 6B). To further observe whether LNT induced cell death, trypan blue dye-exclusion assay was also performed, and the results demonstrated that LNT effectively reduced living cell number (see Fig. S2A), that is, LNT inhibited Hela cell proliferation in a dosage- and time-dependent manner. However, cell death was not observed. Similar to the MTT assay, LNT did not affect proliferation of the normal cell H8 (Fig. S2B). From these data, it can be concluded that LNT specifically inhibited the cell proliferation of tumor cells but not directly killed cells.

### LNT interacts with Hela cells and targets the tumor suppressor p53 to inhibit tumor cell proliferation *in vitro*

Why did LNT suppress the tumor cell proliferation but hardly affect the normal cells *in vitro*? It has been reported that the interaction of polysaccharides with cells is generally considered as the first step in the initiation of some cell responses^37^. To observe if LNT interacts with tumor cells, LNT was conjugated with FITC and incubated with Hela cells followed by confocal microscopy observation. As a result, LNT really bound to the surface of Hela cells indicated by the green fluorescence surrounding the cells (Fig. S3). This result suggested that the interaction of LNT with Hela cells possibly initiated some signal transduction interfering with the proliferation of tumor cells.

Then what happened after interaction of LNT with Hela cells? The animal experiments have shown that LNT inhibited S-180 tumor cell proliferation possibly through targeting the tumor suppressor p53. To see whether LNT interfered with p53 or not *in vitro*, proteins in Hela and S-180 tumor cells were extracted and analyzed by western blot. It was found that LNT remarkably enhanced the expression of p53 in Hela (Fig. 6C,D) and S-180 (Fig. 6E,F) cells after a 15 min-incubation in contrast to the control. In addition, as shown in Fig. 6C,D, p21 expression clearly increased in Hela cells after LNT-treatment for half an hour. Similar to the results *in vivo*, LNT really up-regulated the pro-apoptotic protein Bax expression and down-regulated the anti-apoptotic protein Bcl-2 expression in Hela (Fig. 6C,D) and S-180 (Fig. 6E,F) cells. To confirm if it is the target of LNT, p53 was knocked down by transfecting p53 siRNA, a target-specific 19–25 nt siRNA into Hela cells before treatment with LNT. As a result, knockdown sharply decreased p53 expression in contrast to the non-transfected cells (Fig. 6G), suggesting successful deletion of p53 genes. And expressions of p21 and Bax were significantly down-regulated after p53 knockdown (Fig. 6G), demonstrating that p21 and Bax were directly regulated by p53, which is consistent with the reported results^26^. It is interesting that LNT failed to up-regulate Bax and p21 protein expressions in p53 siRNA-transfected Hela cells, further demonstrating that LNT inhibited Hela cell proliferation through at least partially targeting p53 to regulate p21 and Bax. However, Bcl-2 expression was hardly affected in p53 siRNA-transfected Hela cells with or without LNT treatment (Fig. 6G). Moreover, LNT failed in suppression of cell viability of Hela cells after p53 knockdown (Fig. 6H), also demonstrating that LNT inhibited Hela cell proliferation through targeting p53.

Based on these results, it can be concluded that LNT really interacted with tumor cells, and initiated the action on the p53-dependent pathway, leading to inhibition of tumor cell proliferation *in vitro*. However, herein, it is still unknown which receptors on the surface of cells LNT binds to, which will be further studied in our future work.

### LNT induces activation of caspase 3 *in vitro* tumor cells

Besides, p53 has been shown to induce caspase activation via pro-apoptotic Bcl-2 family proteins^38^. As shown in Fig. 6C (Hela) and **E** (S-180), caspase 3 activation showed a visible enhancement in LNT-treated tumor cells in a time-dependent manner compared with the control, indicating that LNT inhibited the tumor cell proliferation possibly through caspase signaling pathway. However, it was discovered that knockdown of p53 hardly affected the enhancement of caspase 3 activation by LNT (Fig. 6G), suggesting that caspase protein activation regulated by LNT was possibly through the traditional nuclear DNA damage pathways^39^ but not p53-dependent pathway.

## Discussion

LNT, a β-(1, 3)-glucan with β-(1, 6) branches from the fruiting bodies of *Lentinus edodes*, has been shown to be used in cancer therapy combined with chemical drugs in Asia countries, especially in Japan and China. The clinical investigation demonstrated that LNT has low cytotoxicity to normal cells and no obvious side effects in patients^10^. However, so far, the mechanism of anti-tumor action for LNT is still not completely understood. The purpose of this study was thus to get more solid details on its anti-tumor effect and the underlying mechanism. As a result, LNT significantly inhibited S-180 tumor growth *in vivo* (Fig. 1A), and protected the immune system including thymus and spleen from damage in contrast to the positive control of Cytoxan (Table 1 and Fig. 1B, spleen panel). The *in vitro* data also confirmed the non-cytotoxicity against normal cells of H8, LO2 and 293T (Figs 6A and S2B), indicative of a potential candidate for LNT as an anti-cancer/tumor drug.

Since LNT showed remarkable suppression of S-180 tumor growth *in vivo*, it triggered us to investigate how LNT showed the anti-tumor action. As shown in the above results (Figs 1C and 3A, Ki67 panel; Figs 1B and 3B, TUNEL), LNT repressed tumor growth through inhibiting cell proliferation and inducing cell apoptosis. From Fig. 3, it can be seen that T cells and macrophages were recruited into tumor tissues during tumor progression in the control mice, and LNT sharply promoted accumulation of these immune cells, suggesting that LNT activated immune responses. Moreover, significant inhibition of tumor cell proliferation was observed on day 6 (Fig. 3A, Ki67) and the beginning of apoptosis was initiated on day 8 (Fig. 3B, right histogram), demonstrating that immune cells have already existed in the tumor tissue for suppressing cell proliferation and inducing cell apoptosis before cell death. Immune cells are the major source of cytokines/chemokines, and cytokine production acts as a means of communication between both cells and tissues. Herein, TGF-β, a cytokine exerting its tumor-suppressive role by inducing cell-cycle arrest and apoptosis^33^, was found to be significantly enhanced by LNT in tumor tissues in contrast to the positive (Cytoxan) and negative (tumor) controls (Fig. 2D), which was further confirmed by the enhancement of TAK1 expression (Fig. 2E). Besides, IL-1β, IP-10, M-CSF and TREM-1 levels in tumor tissues were also enhanced by LNT (Fig. 2C), further confirming the activated immune responses by LNT in mice. Taken together, LNT inhibited tumor development through activating immune responses to suppress cell proliferation and induce cell apoptosis.

In many human cancers including sarcomas, breast and others, p53, a highly connected “node” in its network, is now recognized to be the most frequently inactivated gene due to degradation of p53 stimulated by extra MDM2 genes or mutation in the DNA-binding domain preventing p53 from binding to specific DNA sequences^40^. Additionally, the recent studies investigating the tumor-suppressive function of p53 demonstrated that the most characterized p53 target is crucial for DNA damage-induced cell cycle arrest, senescence, or apoptosis^41^,^42^,^43^,^44^. Furthermore, one of the first effect of p53 expression, in nearly all mammalian cell types, has been reported to directly stimulate p21 expression for blocking the cell-division cycle^42^. Vogelstein *et al*. also reported that p21 is an inhibitor of cell cycle, which is directly regulated by p53 to block the cell-division cycle^26^. In this work, a sharp up-regulation of p53 and p21 in LNT-treated mice was observed (Fig. 1D,E), indicating that LNT inhibited S-180 tumor growth through blocking cell proliferation possibly via targeting p53. The *in vitro* results showed cell proliferation was suppressed by LNT for Hela and S-180 tumor cells (Figs 6B and S2A). Moreover, LNT up-regulated the expression of p53 protein in Hela and S-180 cells (Fig. 6C–F). Interestingly, LNT also up-regulated p21 expression in Hela cells but failed after p53 siRNA transfection (Fig. 6G). Thereafter, LNT could not inhibit Hela cell proliferation (Fig. 6H), clearly confirming that LNT inhibited tumor cell proliferation through up-regulation of p21 resulting in blocking of tumor cell-division cycle via targeting p53. Of course, this mechanism of anti-tumor cell proliferation occurred *in vitro*. Would it occur *in vivo*? It has been reported that the water-soluble glucans can be absorbed into blood^45^. Therefore, it is possible that LNT samples can be recruited to the tumor site through blood circulation and directly interact with tumor cells to target p53 for suppressing cell proliferation.

It has been reported that some cells in which p53 is activated undergo apoptosis^44^. Bax, an apoptosis-inducing member of Bcl-2 protein family, has been reported to be one of the potential mediators of p53-induced apoptosis, which is directly activated by p53-binding sites in the regulatory region of the gene^42^. Up-regulation of Bax and down-regulation of Bcl-2 in S-180 tumor tissues (Fig. 1D,E) were respectively observed. Additionally, caspase family often functions as vital components of the apoptotic machinery and acts to destroy specific target proteins which are critical to cellular longevity^46^. The active caspases 3 and 9 were also observed (Fig. 1D,E), which were proteolytically generated during apoptosis from the inactive caspase precursors^47^,^48^ as one of the hallmarks of the apoptotic process^49^. Therefore, LNT induced tumor cell apoptosis possibly through p53- and caspase-dependent signaling pathways *in vivo*. As stated above, cleavage of PARP1 by caspase 3 is actually the marker of cell apoptosis^30^ *in vivo*. Down-regulation of PARP1 due to cleavage in LNT-treated mice really occurred (Fig. 1B,C), demonstrating that LNT induced tumor cell apoptosis through caspase-dependent signaling pathway *in vivo*. Moreover, the *in vitro* data demonstrated that caspase 3-dependent pathway was not regulated by p53 (Fig. 6G) but possibly through the traditional nuclear DNA damage pathways^41^. It is interesting that LNT mainly induced inhibition of cell proliferation but not cell apoptosis or death (Fig. S2A) despite high expression of caspase 3 proteins *in vitro* tumor cells (Fig. 6C,E). It has been reported that PARP1 can be cleaved by ICE-like caspases^50^ but not caspase 3 *in vitro*. Therefore, the up-regulation of caspase 3 *in vitro* is not necessary for cell apoptosis, and it is possible that PARP1 was not cleaved *in vitro*, leading to no occurrence of cell apoptosis.

Tumor-induced angiogenesis is critical for the growth of solid tumors^51^,^52^. Recently, anti-angiogenic activities have been increasingly reported in a number of polysaccharides from different sources, such as *Grateloupia longifolia* polysaccharides^53^. As shown in Fig. 4, LNT remarkably reduced the formation of microvessels indicated by the decrease of a marker CD31 of angiogenesis and VEGF in tumor tissues, demonstrating that LNT could act as an inhibitor of angiogenesis to inhibit tumor growth *in vivo*. The anti-angiogenesis induced by LNT was also possibly through activating immune responses to suppress phosphorylation of Stat 3 (Fig. 5), which will be further studied in our future work.

**Figure 7:**
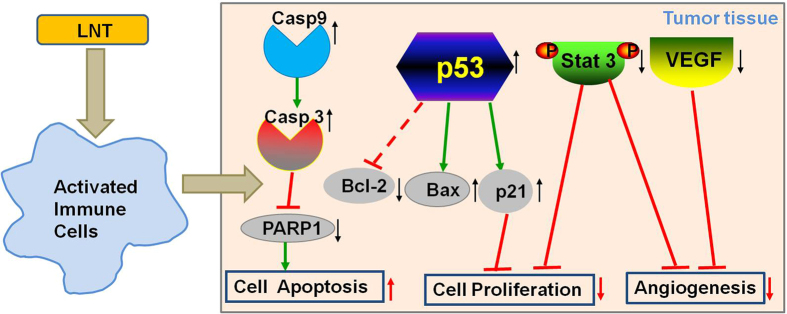
The mechanism map of effects of LNT on cancer cells. LNT activated the immune system to promote tumor cell apoptosis through caspase 3-dependent signaling pathway, and inhibited tumor cell proliferation possibly through targeting p53 via enhancement of p21. In addition, LNT inhibited angiogenesis to suppress tumor growth.

In summary, all the experimental findings strongly demonstrated that LNT showed significant anti-tumor effects without toxicity through activating immune responses to inhibit tumor cell proliferation and angiogenesis, and to induce tumor cell apoptosis *in vivo* (Fig. 7). LNT promoted cell apoptosis through caspase 3-dependent signaling pathway, and inhibited cell proliferation possibly through targeting p53 via enhancement of p21. Additionally, LNT inhibited Stat 3 phosphorylation in tumor tissues, possibly leading to suppression of cell proliferation and angiogenesis. These provide a strong rationale for using LNT to enhance treatment efficacy of cancer.

## Methods

### Sample preparations

The dried fruiting bodies of *Lentinus edodes* were purchased from the supermarket, a commercial product cultivated in Fujian Province of China. The detailed procedure of extraction was slightly modified according to the previously reported method^54^. In brief, the fruiting bodies were immersed in hot water (120 °C, 0.5 h, three times) followed by 1.25 M NaOH/0.05% NaBH_4_ (room temperature, two times). The supernatant after centrifugation to remove the residues was precipitated with 1 M acetic acid to remove α-(1→3)-D-glucan, and was then subjected to the 717 type anion exchange resins to remove proteins, followed by treatment with 30% H_2_O_2_ to decolorize the solution. It was then exhaustively dialyzed by using regenerated cellulose tube (*M*_w_ cutoff 8000) against distilled water for 7 days and ultrapure water for 3 days, finally filtered and concentrated by rotary evaporator at reduced pressure below 44 °C before precipitation into acetone. The precipitates were re-dissolved in pure water and lyophilized to obtain white pure flakes coded as Lentinan (LNT) with a yield of 2.6%.

The total sugar content of LNT was determined to be 98.7% ± 1.53% by the phenol-sulfuric acid analysis using D-glucose as standard^55^, and protein was analyzed to be 0.52% ± 0.07% by the method of Bradford using bovine serum albumin (BSA, Sigma) as the standard^56^, indicating that LNT used in the following experiments was of high purity. The endotoxin was determined by the chromogenic limulus amebocyte lysate assay^57^. It was found that there was no detectable level of endotoxin in LNT samples. LNT was identified as a β-(1, 3)-glucan with β-(1, 6)-branches by GC-MS and NMR consistent with our previous results^9^. The weight-average molecular weight of LNT was determined to be 7.3 × 10^5^ by laser light scattering. LNT was dissolved in the complete cell culture medium or PBS for *in vitro* assays and in normal saline for *in vivo* assays, which was sterilized at 121 °C for 20 min and kept at 4 °C before use.

### Cell culture

The normal human cervical epithelial cell line (H8) was obtained from Shanghai Hong biotechnology co., LTD (Shanghai, China). Human renal epithelial cell line (293T), human cervical carcinoma cell line (Hela), human hepatoyte cell line (LO2) and Mouse sarcoma 180 cell line (S-180) were purchased from China center for typical culture collection (CCTCC). H8, 293T and Hela cells were cultured in Dulbecco’s Modified Eagle’s Medium (DMEM, high glucose, HyClone), while LO2 and S-180 cells were cultured in the altered RPMI 1640 medium (HyClone). All cell lines were incubated in the respective culture medium supplemented with penicillin (100 U/mL), streptomycin (100 μg/mL) and 10% heat-inactivated (56 °C, 30 min) FBS (Gibco, US) at 37 °C under a humidified atmosphere of 95% air and 5% CO_2_.

### Cell viability/proliferation assay *in vitro*

Cell viability was determined by using a standard 3-(4, 5-dimethyl-2-thiazolyl)-2, 5-diphenyl-2-H-tetrazolium bromide (MTT) assay^58^. Tumor cells (Hela and S-180 cells) were seeded in 96-well plates at a concentration of 5 × 10^3^ cells/well in a final volume of 200 μL and incubated for 24 h. The cells were then incubated with LNT dissolved in complete cell culture medium or PBS at final concentrations of 10, 50, 100, 200, 300 and 400 μg/mL. After 48 h incubation, 20 μL of MTT (5 mg/mL, Sigma) solution was added to each well and incubated for 4 h at 37 °C with 5% CO_2_. The supernatant was then removed, and the cells treated with 150 μL of dimethyl sulfoxide (DMSO, Sinopharm, China), followed by gently shaking. The plates were analyzed at a wavelength of 492 nm on a microplate reader (BMG LABTECH, FLUOstar OPTIMA, Germany). The data was presented as cell viability (%) = Experimental group/Negative control group × 100%.

### Tumor challenge and treatment

6 to 8-week-old female and male Balb/c wild-type (WT) mice were purchased from Center for Animal Experiment ABSL-III Laboratory of Wuhan University (Hubei, China). Mice were maintained under controlled conditions of humidity (60 ± 5%), lighting (12 h light cycle) and temperature (24 ± 2 °C) at the Central Laboratory Animal Institute of Wuhan University, and all animal experiments were performed in accordance with national and institutional guidelines for animal care.

S-180 cells were maintained in ascitic form by serial transplantation every seven days in a male mouse. Mice were subcutaneously inoculated with S-180 cells (3 × 10^6^ cells, 0.2 mL) into the right armpit. After inoculation for 24 h, mice were daily treated with i.p. injection of saline (negative control), LNT (1 mg/kg, 5 mg/kg, and 20 mg/kg) and Cytoxan (20 mg/kg, positive control, Sigma) for 10 days. The mice were then followed for tumor growth and sacrificed by cervical dislocation on day 10 for dissection of tumors and lymphoid organs including spleen and thymus. The inhibition ratio against tumor was calculated as ((Average tumor weight of the control group − Average tumor weight of the test group)/Average tumor weight of the control group) × 100%. Thymus and spleen indexes were expressed as the thymus and spleen weight relative to the body weight.

In addition, 6 to 8-week-old male Balb/c wild-type (WT) mice were purchased from Hunan slack scene of laboratory animal co., LTD (Hunan, China). S-180 cells were maintained in ascitic form by serial transplantation every seven days in a male mouse. Mice were subcutaneously inoculated with S-180 cells (3 × 10^6^ cells, 0.2 mL) into the right armpit. 20 Mice were randomized into two groups (n = 10) and received a daily intraperitoneal injection of 0.9% NaCl (control group) and 1 mg/kg of LNT per day in a 200 μL volume once a day after inoculation for 24 h. The mice were then sacrificed by cervical dislocation on day 4, 6, and 8 for dissection of tumors.

### Histology and immunohistochemical staining

Solid tumors and spleens from the control, Cytoxan-, and LNT-treated mice were fixed with 10% phosphate buffered formalin, processed and embedded in paraffin. The sections were then dewaxed and stained with hematoxylin and eosin Y (H&E) for histology observation under a light microscope at 10 × magnification. Immunohistochemistry was performed according to the manufacturer instructions (LSAB kit; Dako, Carpinteria, CA, USA). In brief, tumor sections were cut and deparaffinized in xylene, and dehydrated in graded alcohol and finally hydrated in water. Antigen retrieval was performed by boiling the slide in 10 mM sodium citrate (pH 6.0) for 30 min. Then, slides were incubated overnight with primary antibodies including anti-Bcl-2, anti-Bax, anti-Ki67, anti-CD3, anti-CD68, anti-VEGF, anti-CD31, and anti-phospho-Stat 3 (each at 1:100 dilution). Apoptotic cells were detected using the terminal deoxynucleotidyl transferase-mediated dUTP nick end labeling (TUNEL) apoptosis detection kit (Millipore, USA) according to the manufacturer’s instruction. These slides were subsequently washed several times in Tris-buffered saline with 0.1% Tween-20 and incubated with biotinylated linker for 30 min, followed by incubation with streptavidin conjugate provided in LSAB kit (Dako) according to the manufacturer’s instructions. Immunoreactive species were detected using 3, 3-diaminobenzidine tetrahydrochloride as a substrate. Sections were counterstained with Gill’s haematoxylin and mounted under glass coverslips. Images were taken using a microscope (NIKON ECLIPSE TI-SR, Japan). For quantitation, images were acquired from three different fields at 20 × magnification, and cells were counted using Image pro-plus 6.0 software.

### Immunofluorescence staining

Cryosections of tumors were stained with anti-CD31 (eBioscience) and anti-phospho-Stat 3 (Santa Cruz) antibodies, respectively, followed by a biotinylated secondary antibody (Santa Cruz) and streptavidin-FITC (fluorescein isothiocyanate) with 4′,6-diamidino-2-phenylindole (DAPI; Invitrogen) counterstaining to detect tumor vasculature. The fluorescence images were taken by a microscope (NIKON ECLIPSE TI-SR, Japan), and processed by using Image pro-plus 6.0 software.

### Western blot analysis

The total proteins of Hela and S-180 cells were extracted according to the following procedures. Hela or S-180 cells were plated in a 60-mm culture dish at a density of 3 × 10^6^ cells/dish in the respective culture medium with 10% FBS for 24 h. The cells were then treated with or without LNT under different conditions. At the end of treatment, the cells were harvested and treated with RIPA buffer (50 mM Tris-HCl, 150 mM NaCl, 1% NP-40, 0.5% sodium deoxycholate, 0.1% SDS, and 1 mM EDTA, pH 7.4, Beyotime Institute of Biotechnology) on ice for 20 min. After a 5 min centrifugation at 14000 rpm, the supernatant was saved as the total protein extract. The S-180 tumor tissues were cut into small pieces and lysed by the same RIPA buffer as described above on ice for 20 min. After a 5 min centrifugation at 14000 rpm, the supernatant was harvested as the tumor tissue protein extract. Both cell and tumor tissue proteins were preserved at −70 °C for western blot assay. The protein concentration was determined using a BCA protein assay Kit (Beyotime Institute of Biotechnology).

The cell or tumor tissue lysates were mixed with 4 × SDS sample buffer and denatured in boiling water for 5 min. Aliquots of 20 ~ 40 μg of denatured total proteins were separated by SDS-PAGE on a 12% or 10% polyacrylamide gel and then electrically transferred onto a PVDF membrane (0.45 μm, Millipore). After blocking with 5% (w/v) BSA (Baitg, Wuhan) in TBS (10 mM Tris-HCl (pH 8.0) and 150 mM NaCl) containing 0.1% Tween 20 at room temperature for 1 h, the membranes were then incubated with an appropriate specific primary antibody (Bcl-2 and Bax antibodies from Abcam, p53, caspase 3, caspase 9, PARP1, p21 (C-19), VEGF (147) Stat3 (K-15), phospho-Stat3 (Ser727) and TAK1 (M-579) from Santa Cruz Biotechnology) overnight at 4 °C. To ensure that equal amounts of protein had been loaded into each lane of the SDS gel, antibodies against actin (I-19, goat polyclonal from Santa Cruz Biotechnology, each at 1:200 dilution) were used as loading controls. The reactive bands were visualized with a horseradish peroxidase (HRP)-conjugated secondary antibody (Biosharp) for 50 min via enhanced chemiluminescence (ECL, Baitg) western blot detection reagent on a ChemiDoc-It^TM^ imaging system (UVP, America) according to the manufacturer’s instructions. The images were quantified by densitometric analysis using the UVP-visionworks LS- software.

### Knockdown of p53 with siRNA

Hela cells were seeded into a 96-well plate or a 60-mm culture dish at a certain density in DMEM with 10% FBS. After a 24 h pre-incubation, the cells were transfected with p53 siRNA (Santa Cruz Biotechnology) using Lipofectamine 2000 (Invitrogen) according to the manufacturer’s protocol. After a 12 h transfection, the cells were rinsed with PBS and incubated in DMEM containing 10% FBS for another 48 h. In 96-well plates, the cells were then incubated with LNT dissolved in complete cell culture medium or PBS at different final concentrations of 0, 10, 50, 100, 200, 300 and 400 μg/mL. After 48 h incubation, cell viability was determined by using MTT assay. In the dishes, the cells were then stimulated with LNT (200 μg/mL) for desired time intervals. At the end of treatment, the cells were rinsed with PBS and harvested for the total protein extraction according to the same procedures as described above. The cell lysates were collected for western blot analysis.

### TGF-β measurement and cytokines arrays

TGF-β in tumor lysates was determined by ELISA (R&D Systems) following the manufacturer’s protocol. The OD values were read on a microplate reader at 450 nm (BMG LABTECH, FLUOstar OPTIMA, Germany). The concentration of cytokines was calculated against the standard curve generated using the recombinant cytokine. Cytokines and chemokines in tumor lysates were quantified using customized membrane-based protein arrays (R&D Systems) according to the manufacturer’s instruction. The signals on the membranes were visualized using the enhanced chemiluminescence staining system (ChemiDoc-It^TM^, USA). The images were quantified by densitometric analysis using the UVP-visionworks LS- software.

### Statistical analysis

All data are presented as the mean ± SD from at least two or three independent experiments unless specified otherwise. Student’s *t* test was performed, and the differences were considered statistically significant at *p* < 0.05.

### Ethics statement

The methods were carried out in accordance with the approved guidelines. The Animal Care and Use Committee of Wuhan University (Wuhan, Hubei, China) approved all the animal experiments.

## Additional Information

**How to cite this article**: Xu, H. *et al*. Anti-tumor effect of β-glucan from *Lentinus edodes* and the underlying mechanism. *Sci. Rep.* **6**, 28802; doi: 10.1038/srep28802 (2016).

## Supplementary Material

Supplementary Information

## Figures and Tables

**Table 1:** Anti-tumor activity of the samples against S-180 tumor grown in BLAB/c mice.

Samples	Dose (mgkg^−1^day^−1^)	Days	Inhibition ratio (%)	Enhancement ratio of body weight (%)	Spleen index (%)	Thymus index (%)
Control		10		97.47 ± 1.39	8.87 ± 1.59	1.07 ± 0.083
Cytoxan	20	10	54 ± 8.10^a^	90.88 ± 0.97	5.47 ± 1.26^a^	0.44 ± 0.56^a^
LNT	1	10	75 ± 1.53a,^b^	100.85 ± 0.00	8.43 ± 2.06	1.08 ± 0.427
	5	10	59 ± 9.13^a^	100.59 ± 0.89	9.02 ± 1.64	0.94 ± 0.194
	20	10	68 ± 7.44^a^	103.66 ± 0.03	9.62 ± 1.09	1.42 ± 0.252

Mice were injected with S-180 cells (3.0 × 10^6^ cells/animal s.c.). The animals were treated with the samples, after tumor implantation for 24 h, for ten consecutive days. Data are presented as mean ± SD of ten animals.

^a^*p* < 0.05 compared with Control group by ANOVA followed by student’s t-test.

^b^*p* < 0.05 compared with Cytoxan group by ANOVA followed by student’s t-test.
