# Supplementary material for: Anti-tumor effect of β-glucan from Lentinus edodes and the underlying mechanism
Source: Sci Rep. 2016 Jun 29;6:28802. doi: 10.1038/srep28802 (PMC4926123; doi:10.1038/srep28802)
Supplement: Supplementary Information [file srep28802-s1.pdf]

Supporting Information for

Anti-tumor effect of β-glucan from *Lentinus edodes* and the underlying mechanism

Hui Xu, Siwei Zou, Xiaojuan Xu, Lina Zhang

College of Chemistry and Molecular Sciences, Wuhan University, Wuhan 430072, China

To whom Correspondence should be addressed: Prof. Xiaojuan Xu, College of Chemistry and Molecular Sciences, Wuhan University, Wuhan 430072, Tel/Fax: +86 27 68754188. E-mail: xuxj@whu.edu.cn

Methods

Cell proliferation assay in vitro. For the cell proliferation assay, H8 and Hela cells were seeded in 96-well plates at a density of 6×10^3 cells/well and incubated with various concentrations (0~400 μg/mL) of LNT for 24 h, 48 h and 72 h. Cell proliferation was measured by counting the total number of living cells with Hemocytometer. Cell viability was also observed by the trypan blue dye-exclusion assay. All experiments were performed in triplicate.

Synthesis of FITC conjugated LNT and confocal microscopy. The synthetic method of LNT labeling to the fluorescein isothiocyanate isomer I (FITC, Sigma, US) which covalently reacts with hydroxyl groups is according to the reported procedure[1]. Briefly, LNT (200 mg), FITC (30 mg), pyridine (100 μL，Sinopharm，China) and dibutyltin dilaurate (20 μL，Sinopharm，China) were first dissolved in DMSO (20 mL，Sigma, US). The reaction mixture was heated for 4 h at 100 ℃ and precipitated with 4 volumes of ethanol by centrifugation (6000 rpm, 10 min). The precipitations were repeated four times in total to remove the unbound FITC. LNT labeled with FITC coded as FITC-LNT was finally obtained after drying at 60 ℃.

References

1. Tromp, R. H., van de Velde F., van Riel, J. & Paques, M. Confocal scanning light microscopy (cslm) on mixtures of gelatine and polysaccharides. *Food Res. Int.* **34**, 931-938 (2001).

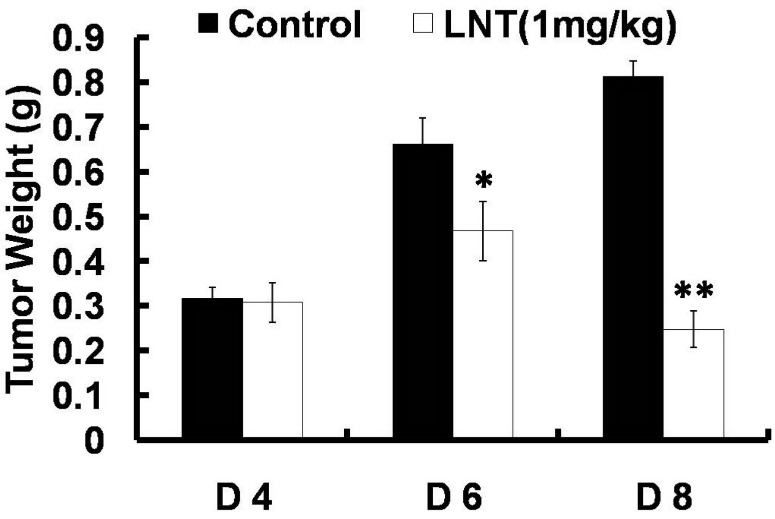

Supplementary Figure 1 Effect of LNT on S-180 tumor weight in mice at day 4, 6, 8; *p< 0.05, **p< 0.001 versus control (n=3, each group).

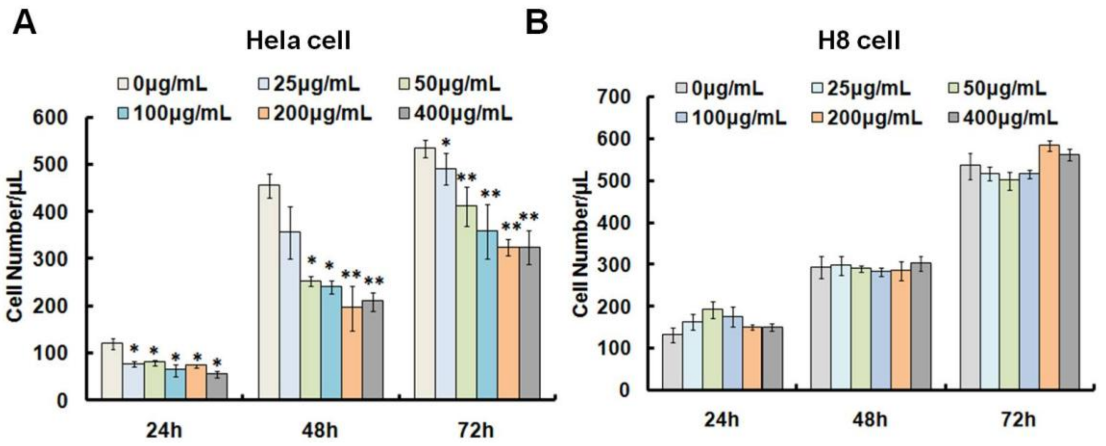

Supplementary Figure 2 Effect of LNT on cell proliferation assay in Hela and H8 cells. (A) Hela Cell and (B) H8 cell proliferation was measured by counting the total number of cells. Cells were seeded in 96-well plates at a density of 6×10^3 cells/well and incubated with various concentrations (0 μg/mL to 400 μg/mL) of LNT for 24 h, 48 h and 72 h. *p<0.05 and **p＜0.001 versus control (0 μg/mL).

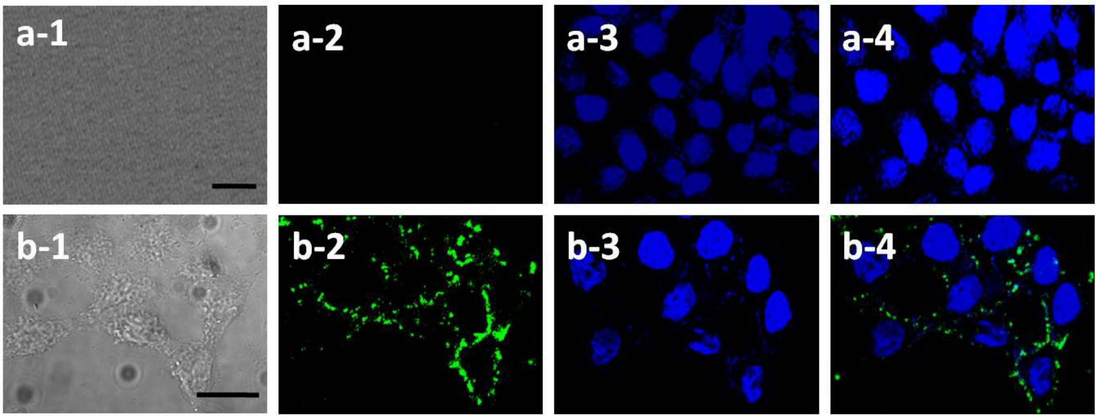

Supplementary Figure 3 Confocal images of Hela cells after incubation with or without LNT-FITC. (a) Blank; (b) LNT-FITC. From left to right: (1) transmitted light images, (2) LNT-FITC images, (3) nucleus dye (Hoechst 33342) and (4) overlapped images of (2) and (3). The blue and green colors correspond to nucleus dye (Hoechst 33342) and LNT labelled FITC, respectively. The micrographs were obtained at a magnification of 600, and the scale bars are 25 μm.
